# Single-Cell RNA Sequencing Defines the Regulation of Spermatogenesis by Sertoli-Cell Androgen Signaling

**DOI:** 10.3389/fcell.2021.763267

**Published:** 2021-11-15

**Authors:** Congcong Cao, Qian Ma, Shaomei Mo, Ge Shu, Qunlong Liu, Jing Ye, Yaoting Gui

**Affiliations:** ^1^Guangdong and Shenzhen Key Laboratory of Male Reproductive Medicine and Genetics, Peking University Shenzhen Hospital, Institute of Urology, Shenzhen-Peking University-The Hong Kong University of Science and Technology Medical Center, Shenzhen, China; ^2^Department of Reproductive Medicine, Peking University Shenzhen Hospital, Shenzhen, China

**Keywords:** spermatogenesis, AR, scRNA-seq, male infertility, knockout

## Abstract

Androgen receptor (AR) signaling is essential for maintaining spermatogenesis and male fertility. However, the molecular mechanisms by which AR acts between male germ cells and somatic cells during spermatogenesis have not begun to be revealed until recently. With the advances obtained from the use of transgenic mice lacking AR in Sertoli cells (SCARKO) and single-cell transcriptomic sequencing (scRNA-seq), the cell specific targets of AR action as well as the genes and signaling pathways that are regulated by AR are being identified. In this study, we collected scRNA-seq data from wild-type (WT) and SCARKO mice testes at p20 and identified four somatic cell populations and two male germ cell populations. Further analysis identified that the distribution of Sertoli cells was completely different and uncovered the cellular heterogeneity and transcriptional changes between WT and SCARKO Sertoli cells. In addition, several differentially expressed genes (DEGs) in SCARKO Sertoli cells, many of which have been previously implicated in cell cycle, apoptosis and male infertility, have also been identified. Together, our research explores a novel perspective on the changes in the transcription level of various cell types between WT and SCARKO mice testes, providing new insights for the investigations of the molecular and cellular processes regulated by AR signaling in Sertoli cells.

## Introduction

Spermatogenesis is a precise process in mammals, including mitosis of spermatogonial, meiosis of spermatocyte and spermiogenesis. As the only somatic cells in seminiferous tubules, Sertoli cells play an important role in structure, nutrition and regulation (Griswold, 2018). Previous studies showed that in Sertoli cells, androgens could bind to androgen receptors (AR), which facilitated the binding of AR to the androgen response element (ARE) and the recruitment of coregulators to induce target gene transcriptional activation or repression (Heinlein and Chang, 2002; Nantermet et al., 2004; Wang et al., 2009; Smith and Walker, 2014). Mutations in AR failing to activate its target genes result in hereditary disorders such as androgen insensitivity syndrome (AIS) or testicular feminization (*Tfm*) mutation in human (La Spada et al., 1991; Griffin, 1992; Quigley et al., 1995; McPhaul, 1999). Similar female-typical external appearance in a human with CAIS or *Tfm* mouse was also observed in the male total AR knockout (T-AR^–/*y*^) mice (Yeh et al., 2002; Matsumoto et al., 2003). In addition, other studies have revealed that Sertoli cell-specific AR knockout (SCARKO) mice were infertile, with spermatogenesis arrest at the diplotene stage of the first meiosis. The number of Sertoli cells and spermatogonia was not affected, spermatocytes were slightly decreased, yet post-meiotic spermatids were remarkably reduced or even absent (Chang et al., 2004; De Gendt et al., 2004; Holdcraft and Braun, 2004). These results further stressed the vital role of AR signaling for both external and internal male phenotype development.

Although the importance of androgen action in the development and function of testicular seminiferous tubules has been clearly evidenced, the mechanisms underlying still remain unclear. In recent years, many groups have used selective or global AR knockout mouse models to search for AR regulated target genes in Sertoli cells. A first AR-knockout study compared the difference of transcript levels in testes from 10-day-old SCARKO and control mice by microarray analysis, and unexpectedly at least 692 genes were found differentially expressed on day 10 (Denolet et al., 2006b). Forty of them showed at least twice the difference (28 down-regulated and 12 upregulated in the SCARKO). In our previous study, we studied the testosterone/AR regulated genes by comparing the gene expression difference in SCARKO mice testis and TM4 cell line stably expressing AR (TM4/AR) with testosterone treatment. 603 differentially expressed genes (DEGs) were identified in the overlap part, including 164 upregulated and 439 downregulated by androgen and AR (Zhang et al., 2012). Although some target genes regulated by AR have been reported, there is very limited overlap between the sets of androgen-regulated genes identified in the available studies. Therefore, it is of great academic significance to continue to search for the target genes directly affected by AR in Sertoli cells of testis and determine the mechanism of action of these target genes for further elucidating the molecular mechanism of AR regulating spermatogenesis.

Single-cell RNA sequencing (scRNA-seq) is a new technology for high-throughput sequencing analysis of the transcriptome and epigenome at the single-cell level. It reveals the gene expression profile of single cells, and reflect the heterogeneity between cells (Gawad et al., 2016; Stuart and Satija, 2019). Many studies have focused on spermatogenesis and studied different cell populations in testis at high resolution by using scRNA-seq (Chen et al., 2018; Wang et al., 2018). Most recently, an scRNA-seq analysis of testicular transcriptomes in SCARKO mice showed a block in the transcriptomes of germ cells in an early meiotic prophase state and identified several mis-regulated genes in Sertoli cells (Larose et al., 2020). In addition, they confirmed that Sertoli-cell AR signaling was dispensable for early meiotic prophase events. However, the cellular heterogeneity, the major differentiation signals, and cell–cell interactions among these cells in SCARKO mice testis are still unknown. Here, we profiled single-cell transcriptomes of 39411 individual cells from the p20 testes of SCARKO and wild type (WT) mice using scRNA-seq. Focusing on the somatic cell dataset, we observed that the distribution of Sertoli cells was completely different and uncovered the cellular heterogeneity and transcriptional changes in WT and SCARKO Sertoli cells. There were 347 up-regulated genes and 250 down-regulated genes observed in SCARKO Sertoli cells, many of which have been previously implicated in cell cycle, apoptosis and male infertility. In addition, we identified 80 up-regulated and 22 down-regulated genes that were associated with spermatogenesis, reproductive process, male gamete generation and germ cell development in SCARKO germ cells, which suggested that this gene set might function to license spermatocytes for progression toward spermiogenesis. Collectively, our results provide in-depth insight into the spermatogenic microenvironment and the investigations of the molecular and cellular processes regulated by AR in Sertoli cells.

## Results

### Overview of scRNA-Seq in Wild-Type and SCARKO Testes at P20

**FIGURE 1:**
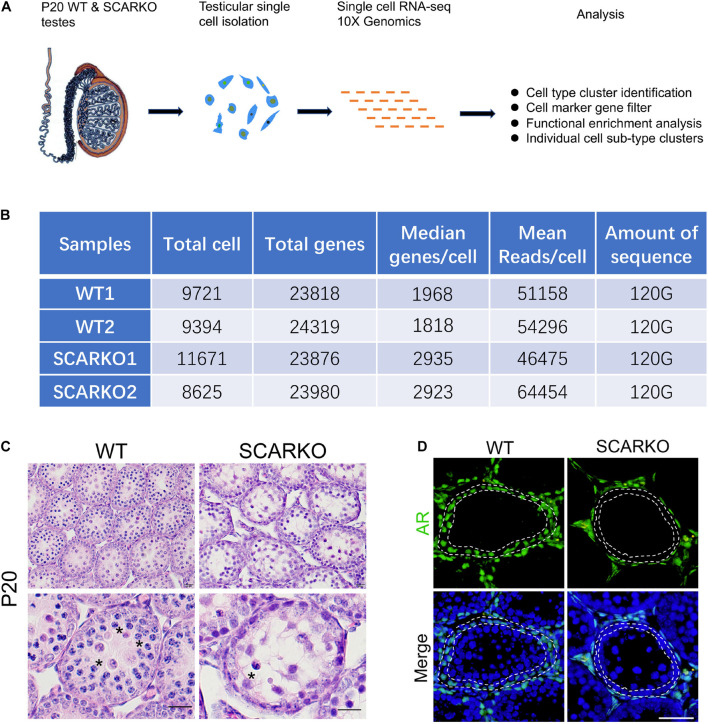
Schematic overview of scRNA-seq using WT and SCARKO testes at P20. **(A)** The sequencing analysis process of scRNA-seq. **(B)** Sequencing metrics after quality control of scRNA-seq datasets. **(C)** HE stanning of the testis from WT and SCARKO mice at P20. Asterisks indicate spermatocyte. Scale bar, 20 μm. **(D)** AR protein expression in WT and SCARKO testes at P20 analyzed by immunofluorescent staining. AR and nuclei were labeled by anti-AR antibody (green) and DAPI (blue), respectively. Scale bar, 20 μm.

To characterize the cellular diversity of testes, we obtained the single cell suspension from four testes of P20 WT and SCARKO mice, respectively, and performed 10X scRNA-seq technique to identify the intercellular relationships involved in spermatogenesis (Figure 1A). After filtering out poor-quality testicular cells, 19115 WT and 20296 SCARKO cells were retained for further analysis. We detected an average of ∼1,900 and ∼2,900 genes per cell in WT and SCARKO mice, respectively, with a total of ∼24,000 genes in all cells, which are sufficient to identify different cell types involved in spermatogenesis (Figure 1B). The hematoxylin-eosin staining results showed that number of meiotic germ cells was significantly reduced and surviving spermatocytes failed to progress in P20 SCARKO testis compared to WT testis, which was consistent with other studies that very few spermatocytes completing the first meiotic division in SCARKO mice testis (11, 12) (Figure 1C). To examine the AR knock out efficiency, we performed the immunofluorescent staining of AR in testis sections, and observed that SCARKO mice resulted in loss of AR-positive Sertoli cells compared with WT mice (Figure 1D).

### Testicular Cell Type Identification and Clustering Analysis in Wild-Type and SCARKO Mice

**FIGURE 2:**
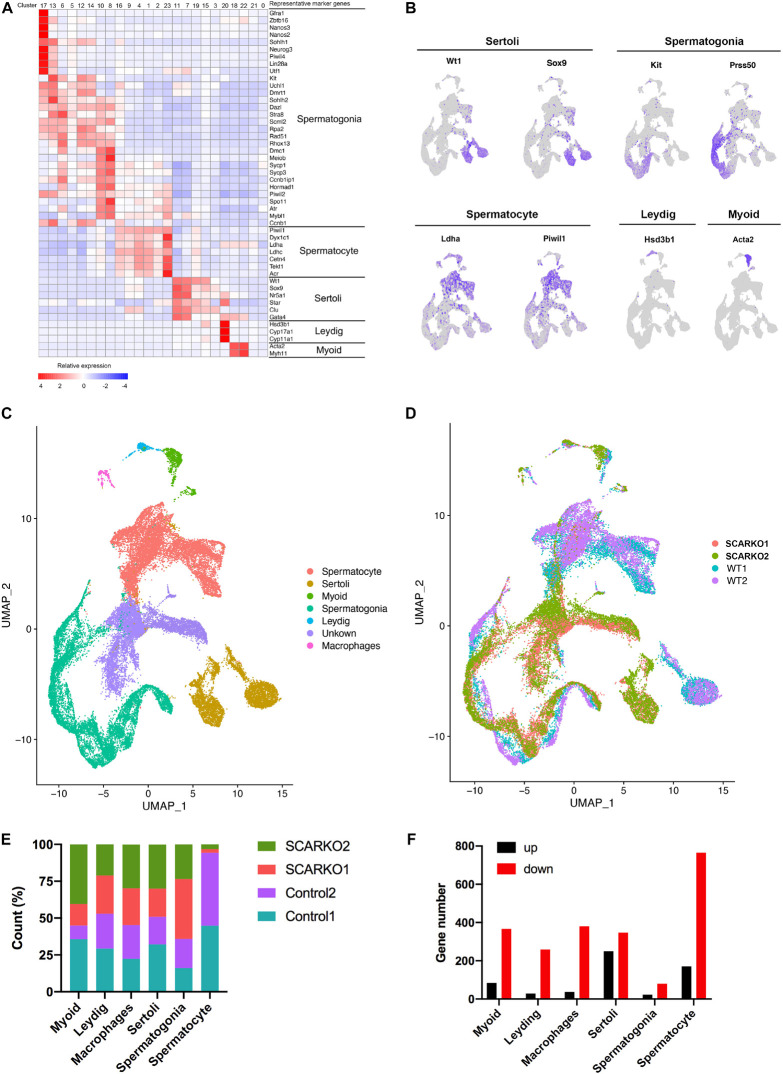
ScRNA-seq profiles of P20 WT and SCARKO testicular cells and cell type identification. **(A)** Heatmap of differentially expressed genes (DEGs) from the five major cell types. Top: cell cluster; right: major cell types and representative enriched marker genes for each cell type. **(B)** Representative markers for each cell type. **(C,D)** tSNE and clustering analysis of combined single-cell transcriptome data from P20 WT and SCARKO testicular cells. Each dot represents a single-cell and cell clusters are distinguished by colors. **(E)** The percentage of cell numbers in each cluster in P20 WT and SCARKO testicular cells. **(F)** The number of DEGs (up and down-regulated) in each cluster in SCARKO testicular cells.

After sequencing, several software packages, including CellRanger, Seurat, PCA, and UMAP were used for the testicular cell cluster and classification analysis. The results showed that there was a very distinct heterogeneity between WT and SCARKO groups, but all testis samples within each group had a good repeatability (Supplementary Figure 1A). Unsupervised clustering of the total 39,411 testicular cells projected onto tSNE analysis plots identified 24 cell clusters (Supplementary Figure 1B). Then the enriched expression analysis of marker genes in each cell cluster are shown in Supplementary Figure 1D. To further identify the cell type of each cluster, we selected the marker genes of each testicular cell type, including four somatic cell populations: Myoid cells, Sertoli cells, Leydig cells, and macrophages and two male germ cell populations: spermatogonia and spermatocyte (Figures 2A–C). In addition, we also identified an unknown cell population expressing some specific genes including Defb19, Fabp9, Uchl1, and Hmgn1 (Supplementary Figure 1D). The expression localization of some marker genes used to define various cell populations are shown in Figure 2B. And AR was primarily expressed in somatic cells including Myoid cells, Leydig cells, and Sertoli cells and was not expressed in germ cells (Supplementary Figure 1C) which was consistent with previous findings in mouse testis (Chang et al., 2004; De Gendt et al., 2004). Biological replicates exhibited similar cell distributions. However, the distribution of Sertoli cells was completely different between WT and SCARKO groups, which might be caused by the deletion of AR in Sertoli cells (Figure 2D).

We determined that 44.9, 52.9, 45.3, 50.9, 35.9, and 94.3% cells sorted into Myoid cells, Leydig cells, macrophages, Sertoli cells, spermatogonia, and spermatocytes, respectively, in WT group and 55.1, 47.1, 54.7, 49.1, 64.1, and 5.7% cells sorted into Myoid cells, Leydig cells, macrophages, Sertoli cells, spermatogonia, and spermatocytes, respectively, in SCARKO group (Figure 2E). There were almost same proportions of each somatic cell type between the two groups, but the percentage of spermatocytes in SCARKO group showed a dramatic decrease suggests that lack of AR in Sertoli cells severely impairs normal spermatogenesis. In addition, analysis of DEGs between WT and SCARKO groups in all cell types revealed that most of genes were downregulated in SCARKO cells especially in spermatocytes (Figure 2F).

### Identification of Sertoli Cell Clusters in Wild-Type and SCARKO Mice

**FIGURE 3:**
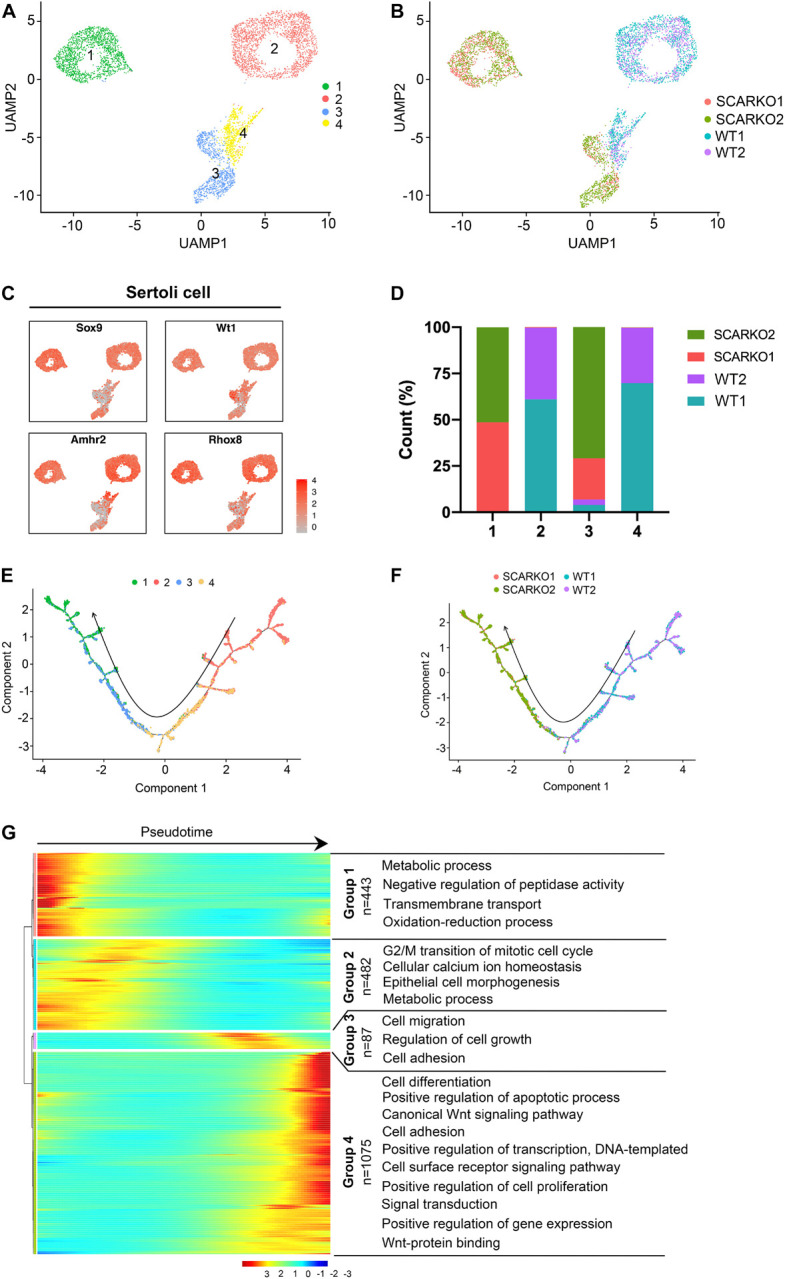
Identification of Sertoli cell clusters in WT and SCARKO mice. **(A,B)** UMAP plot of SCs from P20 WT and SCARKO mice. **(A)** SC clusters; **(B)** sample source. **(C)** Expression patterns of marker genes for SCs visualized in tSNE plots. **(D)** The percentage of cell numbers in each cluster in P20 WT and SCARKO testicular SCs. **(E,F)** Monocle pseudotime trajectory analysis of the SC clusters. **(E)** SC clusters; **(F)** segregated by sample source. Arrow indicates the inferred developmental direction from the analysis shown in **(A)**. **(G)** Heatmap of DEGs from different SC subsets following the trajectory timeline shown in **(E)**. Top: pseudotime directions; right: the number of DEGs and the representative biological processes.

To explore the heterogeneity of Sertoli cells between WT and SCARKO testes and determine how loss of AR effects the transcriptome-wide signatures of Sertoli cells, we re-clustered the 2667 WT and 2576 SCARKO Sertoli cells. Based on analysis of UMAP, four distinct Sertoli cell subtypes were identified (Figure 3A). The heatmap of DEGs in each subtype was shown in Supplementary Figure 2A. And the top Gene ontology (GO) terms for these DEGs in four Sertoli cell subtypes were identified in Supplementary Figures 2C–F. Interestingly, we observed WT and SCARKO Sertoli cells are distributed in completely distinct clusters, suggesting that Sertoli cells undergo a dramatic change after losing of AR signaling (Figure 3B). Marker gene analysis suggests that subcluster 1 and 2 expressed higher levels of Sertoli cell marker genes including Sox9, Wt1, Amhr2, and Rhox5 than that in subcluster 3 and 4 (Figure 3C). Most Sertoli cells in subcluster 2 and 4 were identified as WT cells, and most Sertoli cells in subcluster 1 and 3 were identified as SCARKO cells (Figure 3D). To analyze the origin and maturation process of Sertoli cells, we performed pseudotime trajectory analysis. The result revealed a linear trajectory of Sertoli cells from WT and SCARKO mice (Figure 3E). It is worth noting that Sertoli cells from WT were mainly distributed in the first half of the pseudotime trajectory, while Sertoli cells from SCARKO were almost distributed in the second half of the pseudotime trajectory (Figure 3F). This result suggested that lack of AR in Sertoli cells might affect the development and maturation of Sertoli cells. Finally, we identified four dynamic expression patterns of DEGs along the pseudotime trajectory and the GO terms of these four patterns (Figure 3G). These four groups were revealed to be related with the developmental age of the Sertoli cells, and the DEGs in each group were enriched in various biological processes which represented different stages of Sertoli cells development. Notably, there was a striking shift in transcriptional programs between group 2 and group 3, dominated by expression of cell migration/growth genes, suggesting that this transition may represent a critical function node produced by AR signaling in Sertoli cells.

### Loss of Functional Sertoli Androgen Receptor Affects Cellular Activity and Alters the Transcriptome of Sertoli Cells

**FIGURE 4:**
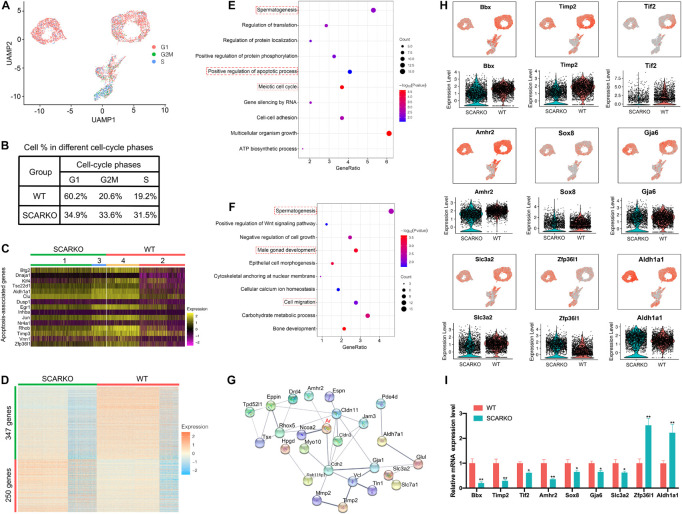
Loss of functional Sertoli AR affects cellular activity and alters the transcriptome of Sertoli cells. **(A)** UMAP plot inferring the cell-cycle phase based on expression of a large set of G2/M- and S-phase genes (47). **(B)** Percentages of SCs in different cell-cycle phases from different WT and SCARKO mice. **(C)** Heatmap of apoptosis-associated genes in SCs of WT and SCARKO mice. **(D)** Heatmap of DEGs between WT and SCARKO Sertoli cells. **(E,F)** GO term analysis of up-regulated **(E)** and down-regulated **(F)** genes in SCs of SCARKO mice. **(G)** Gene regulatory networks of DEGs shown in **(D)**. **(H)** Violin plot showing the expression levels of DEGs in WT and SCARKO SCs. **(I)** qPCR results showing the expression fold change of DEGs showed in **(H)**.

To identify distinct Sertoli cells engaging in proliferation and apoptosis, we first examined the expression pattern of cell cycle specific genes in WT and SCARKO Sertoli cells (Figure 4A). The result showed that the genes related with G2M and S stage had a higher expression level in SCARKO Sertoli cells than that in WT Sertoli cells, suggesting a higher proliferation rate in SCARKO Sertoli cells (Figure 4B). In addition, the expression levels of apoptosis-related genes in SCARKO Sertoli cells were higher than those in WT Sertoli cells (Figure 4C). Our results reveled that Sertoli cells lack of AR exhibited higher proliferative ability and apoptotic rate.

The heterogeneity of Sertoli cells was further confirmed by the heatmap of differentially expressed genes between WT and SCARKO mice (Figure 4D). There were 347 up-regulated genes and 250 down-regulated genes observed in SCARKO Sertoli cells. GO term and KEGG pathway analysis revealed that the up-regulated genes were mainly related to spermatogenesis, positive regulation of apoptotic process and meiotic cell cycle (Figure 4E and Supplementary Figure 2G), while the down-regulated genes were significantly associated with spermatogenesis, male gonad development and cell migration (Figure 4F and Supplementary Figure 2H), indicating lack of AR signaling in Sertoli cells obviously disturbs the function of Sertoli cells. Identification and analysis of DEGs between WT and SCARKO Sertoli cells revealed that 85 of which have been overlapped in the previous publication, for example, Rhox5, Eppin, Tsx, Gpd1, Tpd52l1, and Serpina5 (Supplementary Figure 2B; Zhang et al., 2012). While some of other DEGs might be AR-regulated were first reported in this literature such as Bbx, Timp2, Tif2, Amhr2, Sox8, and Zfp36l1 (Figure 4H). Then we validated the expression levels of several DEGs by RT-qPCR in WT and SCARKO Sertoli cells (Figure 4I). In addition, the DEGs are predicted to form complex gene regulatory networks in Sertoli cells (Figure 4G). In general, these results reveal that lack of AR signaling in Sertoli cells globally influences the expression of genes that control multiple cellular processes, including proliferation, apoptosis and migration of Sertoli cells and spermatogenesis.

### Identification of Four Stages and Heterogeneity of Spermatogonia in Wild-Type and SCARKO Mice

**FIGURE 5:**
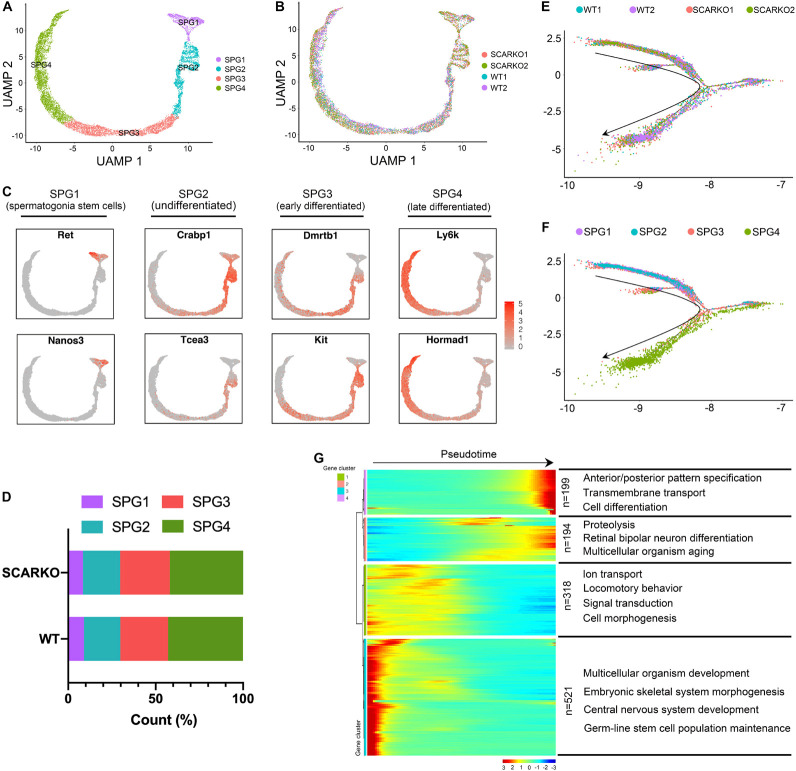
Identification of spermatogonia clusters in WT and SCARKO mice. **(A,B)** UMAP plot of spermatogonia from P20 WT and SCARKO mice. **(A)** spermatogonia clusters; **(B)** sample source. **(C)** Gene expression patterns of selected marker genes corresponding to each cellular state on the UMAP plots. **(D)** The percentage of cell numbers in each state in P20 WT and SCARKO spermatogonia. **(E,F)** Monocle pseudotime trajectory analysis of the spermatogonia clusters. spermatogonia clusters; **(F)** segregated by sample source. Arrow indicates the inferred developmental direction from the analysis shown in **(A)**. **(G)** Heatmap of DEGs from different spermatogonia subsets following the trajectory timeline shown in **(E)**. Top: pseudotime directions; right: the number of DEGs and the representative biological processes.

Due to SCARKO germ-cell arrest occurs at the early meiotic spermatocytes, we next set out to investigate how AR signaling deficiency in Sertoli cells affects the germ cell transcriptional landscape and the ultimate ability of germ cells to acquire meiotic competence. First, we performed re-clustering of 3758 WT and 6711 SCARKO spermatogonial cells and identified 4 subtypes based on expression patterns of known germ-cell marker genes (Figure 5A and Supplementary Figure 3A). None of the subtypes derived solely from WT or SCARKO spermatogonial cells and cells in two groups exhibited similar cell distributions (Figure 5B). The results showed that cells in SPG1 corresponded to spermatogonia stem cells, SPG2 corresponded to undifferentiated spermatogonia, SPG3 corresponded to early differentiated spermatogonia and SPG4 corresponded to late differentiated spermatogonia (Figure 5C and Supplementary Figure 3B). The several top GO terms for DEGs in each of the SPG1-4 states are shown in Supplementary Figures 3C–F. Then we determined that the percentage of each subtype cells between WT and SCARKO groups was comparable, suggesting that AR signaling deficiency in Sertoli cells has almost no impact on germline stem cell development (Figure 5D). Pseudotime analysis provided an arrow vector, which aligned with the developmental order of germline stem cells from SPG1 to SPG4 (Figures 5E,F). Finally, we identified four dynamic expression patterns of DEGs along the pseudotime trajectory and the GO terms of these four patterns (Figure 5G).

**FIGURE 6:**
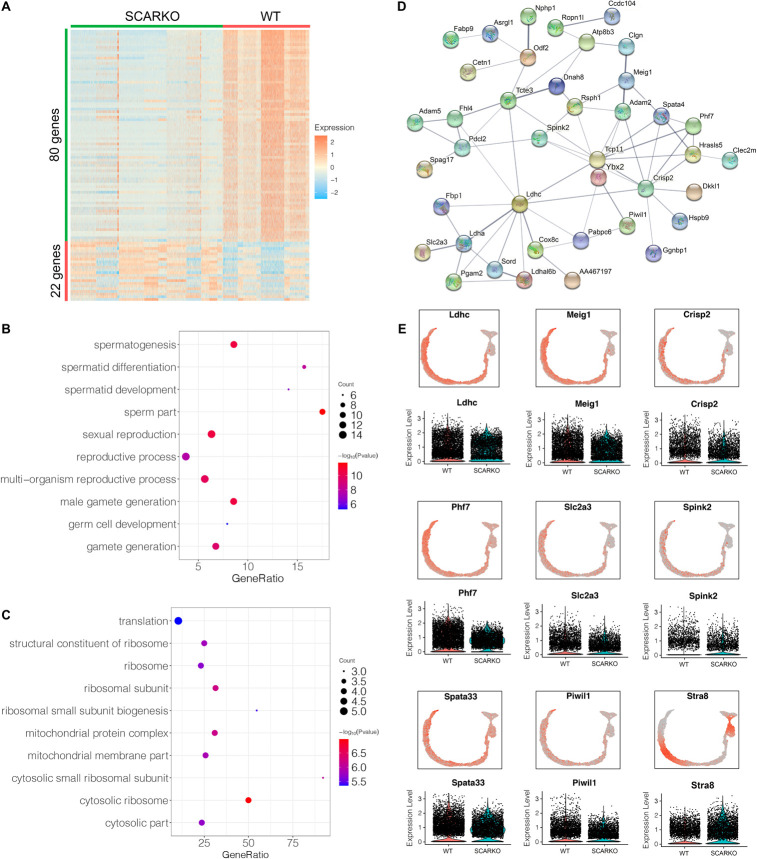
Identification of DEGs in spermatogonia from WT and SCARKO mice. **(A)** Heatmap of DEGs between WT and SCARKO Sertoli cells. **(B,C)** GO term analysis of up-regulated **(B)** and down-regulated **(C)** genes in SCs of SCARKO mice. **(D)** Gene regulatory networks of DEGs shown in **(A)**. **(E)** Violin plot showing the expression levels of DEGs in WT and SCARKO spermatogonia.

Spermatogenic cells undergo ordered and complex changes during spermatogenesis. To study the transcriptional difference of spermatogonia between WT and SCARKO, we detected 22 upregulated genes and 80 down-regulated genes in SCARKO spermatogonia (Figure 6A). GO term and KEGG pathway analysis revealed that downregulated DEGs were significantly related with those that function in spermatogenesis, reproductive process, male gamete generation and germ cell development (Figure 6B and Supplementary Figure 3G). While up-regulated genes were mainly enriched in ribosome and mitochondrial membrane part (Figure 6C and Supplementary Figure 3H). Figure 6D shows a very complex gene regulatory network of DEGs in spermatogonia. Then we validated the expression patterns of the DEGs by visualized in tSNE and violin plot (Figure 6E). The expression levels of some known transcripts (e.g., Ldhc, Spata33, Piwil1, and Stra8) involved in spermatogenesis were significantly changed in SCARKO spermatogonia. In general, these results reveal that lack of AR signaling in Sertoli cells globally influences the expression of genes that control multiple spermatogenic processes.

## Discussion

Spermatogenesis and the interaction between somatic cells and germ cells play a crucial role in male reproduction. During spermatogenesis, testosterone is essential for the maintenance of BTB, completion of meiosis, adherence of elongated spermatids to Sertoli cells as well as the release of sperm. A number of AR regulated genes in SCARKO mice have been identified through gene expression analysis, however, the detection techniques were somewhat mystifying as large changes in gene expression that may lead to response to a hormone essential for spermatogenesis could not be detected (Denolet et al., 2006b; Zhang et al., 2012). In our study, to investigate the transcriptome change in SCARKO mice spermatogenesis and testicular somatic cells, the testicular single cell suspension of WT and SCARKO mice was isolated for scRNA-seq using a 10 × genomics platform. Two male germ cell types and three testicular somatic cell types were clustered, and the DEGs of each cell type were also identified. Our study elucidated the classification of cell types in SCARKO mice testes and analyzed a series of candidate genes that might be regulated by AR and potentially play key roles in spermatogenesis.

Recently, several groups have tried to identify androgen-regulated genes in the testis with androgen-treated neonatal mice (P8), *hpg* mice (P35–45) (Sadate-Ngatchou et al., 2004; Zhou et al., 2005) or SCARKO mice (P10) (Denolet et al., 2006a). Except for *Rhox5*, little overlap of androgen-regulated genes has been found between these studies. There are 409 genes showing significant expression change (82 down-regulated and 327 up-regulated in the androgen-insensitive animal) in both the 10-d SCARKO testes and 20-d testicular feminized (*Tfm)* mice testes although only 7 of these genes show a more than twofold difference (O’Shaughnessy et al., 2007). The probable explanation for this obvious discrepancy is that, previous studies in SCARKO mice have mainly focused on the mixed cells of the testis, and the DEGs analysis in each cell type, including spermatogonial cells, spermatocytes and somatic cells, has not been investigated to date. To explore the DEGs analysis during spermatogenesis between p20 WT and SCARKO mice testes, 10 × genomics scRNA-seq was performed in our research. Testes from mice at 20 days old were chosen because this is the time when Sertoli cells cease proliferation and the process of meiosis starts (Vergouwen et al., 1993; Baker and O’Shaughnessy, 2001), while full spermatogenesis has not developed and the testes have not descended.

In our study, two germ cell types containing spermatogonia and spermatocyte and four types of somatic cell including Sertoli cells, Leydig cells, myoid cells, and Macrophages were identified by scRNA-seq. The results revealed that the number of all types of somatic cells in WT and SCARKO testes showed no significant difference. However, the proportion of spermatogonia in WT was less than that in SCARKO, while the proportion of spermatocyte in WT was over 90%. This was consistent with the phenotype of SCARKO mice that spermatogenesis arrest at the diplotene stage of the first meiosis. Interestingly, we find the distribution of Sertoli cells was completely different in WT and SCARKO mice, which uncovered the cellular heterogeneity and dramatic transcriptional changes after the deletion of AR in Sertoli cells. One consistent finding from our studies and other’s is that a high percentage of genes in Sertoli cells was down regulated in response to testosterone among DEGs in WT and SCARKO testes (Denolet et al., 2006a; O’Shaughnessy et al., 2007). Moreover, other testicular cell types also exhibited high percentage of down-regulated genes in SCARKO mice as shown in our study. This high percentage of down-regulated genes could not be directly regulated by AR. Instead, AR-mediated increase in gene expression might be negative modulators of other genes, or that testosterone signaling may affect the metabolism of Sertoli cells which lead to decrease in gene expression.

To explore the crucial genes which might be regulated by AR or associated with spermatogenesis in testis, we independently re-clustered the Sertoli cells and germ cells from our scRNA-seq datasets. Four patterns of gene expression dynamic along a linear trajectory of SCs were identified through pseudotime trajectory analysis of SCs, which suggested that SCs undergo discrete step-wise shifts in gene expression between the WT and SCARKO mice. GO analysis revealed that many genes involved in “spermatogenesis,” “positive regulation of apoptotic process,” and “meiotic cell cycle” are upregulated, while other genes related to “male gonad development” and “cell migration” are downregulated in SCARKO mice Sertoli cells. This result was consistent with the cell cycle and apoptosis analysis and indicated that the loss of AR function in Sertoli cells promoted cell proliferation and apoptosis.

In addition, 85 DEGs in SCARKO mice have been reported in other studies that might be regulated by AR, which confirms the reliability of our scRNA-seq. However, several other DEGs which have known roles in Sertoli cells development, or cause male infertility when deficient were first reported in our study. For example, Bbx is an evolutionally conserved gene and localized in the nuclei of both Sertoli cells and postmeiotic spermatids in the testis. However, loss of BBX leads to apoptosis of postmeiotic spermatids, spermiogenesis defects and ultimately, male infertility (Wang et al., 2016). Results from the scRNA-seq indicate that androgens could promote Bbx expression, suggesting that inhibition may exert deleterious effects on normal testicular function. TIMP2 is predominantly expressed in Sertoli cells and may participate in testis development via regulating cell migration and tissue remodeling (Grima et al., 1996; Longin and Le Magueresse-Battistoni, 2002; Siu and Cheng, 2004). In our study, TIMP2 showed down-regulated expression in the SCARKO testis. It was reported that the prototypical testicular toxic phthalate monoester, mono-(2-ethylhexyl) phthalate (MEHP), suppresses TIMP2 levels in Sertoli cell, triggers germ cell apoptosis and disrupts the connections between germ cells and Sertoli cells (Yao et al., 2009, 2010). The transcriptional activity of AR is reported to be regulated by an array of coregulators. Among the currently known coregulators, TIF2 was significantly reduced after deletion of AR in Sertoli cells. TIF2 is expressed in Sertoli cells but not in germ cells in male mice. TIF2 ablation in mice caused spermiogenesis defects, malformed spermatozoa, testicular degeneration, and increase in intracellular lipids of Sertoli cells (Gehin et al., 2002; Mark et al., 2004). ZFP36L1 is an RNA-binding protein responsible for cytoplasmic mRNA decay (Varnum et al., 1991; Blackshear, 2002). It contains a potential nuclear localization signal and a nuclear export signal, and might have a potential to shuttle between the nuclei and cytoplasm. There was evidence showed that the nuclear accumulation of ZFP36L1 protein is regulated in a cell cycle-dependent manner and has important roles in cell proliferation (Matsuura et al., 2020). *In vitro* overexpression of ZFP36L1 in SET-2 cell line affected cell proliferation and induced apoptosis (Martínez-Calle et al., 2019), which was consistent with our scRNA-seq analysis that overexpressed ZFP36L1 might affect cell apoptosis and proliferation in SCARKO Sertoli cells. Collectively, analysis of the scRNA-seq from SCARKO testis strengthens the idea that testosterone signaling is essential for spermatogenesis through combined effects on multiple genes or the regulation of protein expression in the testis that may not have effects until well downstream.

Sertoli cells play a critical role in the regulation of germ cell development. At puberty, adjacent SCs form specialized tight junctions at the base, dividing the tubules into a basal compartment mainly containing spermatogonia and an abluminal compartment containing spermatocytes and subsequent stages of germ cell development (Jégou et al., 1983). However, one question about how meiosis is supported by testosterone still needs further investigation. A recent scRNA-seq study –focusing on the SCARKO germ cells– have obtained evidence that the expression and localization of key protein markers of meiotic prophase events was normal in SCARKO mutant spermatocytes, implying that the initiation of meiotic prophase is not androgen dependent (Larose et al., 2020). In our study, we find the cell number of SCARKO spermatocyte was significantly decreased which accounts for less than 10% of the total number, but spermatogonia was comparable between two groups. To profile the transcriptome-wide signatures during germline stem cell development and how AR signaling effects this process, we performed focused re-clustering of WT and SCARKO spermatogonial cells. Our analysis of scRNA-seq data showed that: (1) four spermatogonial states (SPG1-4) follow a continuous differentiation trajectory and that AR deficiency in Sertoli cells doesn’t impair germline stem cell development as evidenced by comparable percentages of differentiated cells in SPG3/4 states; (2) AR signaling deficiency affects germ cell development- and spermatogenesis-related genes in SCARKO spermatogonia.

In summary, our investigations introduce the role of AR in the complex regulatory networks between different testicular cell-cell crosstalk, and AR as a transcription factor that is necessary for proper function of Sertoli cell, spermatogenesis and male fertility. These findings not only provide new insights into molecular mechanisms of Sertoli cell function, but also a better understanding of androgen signaling in Sertoli cells creates a permissive environment for germ-cell progression through meiosis and gamete production.

## Materials and Methods

### Animals

All animal experiments were approved by the ethics committee of Peking University Shenzhen Hospital and animal center of Shenzhen PKU-HKUST Medical Center. C57BL/6 J wild-type (WT) mice and the SCARKO mice which had been described previously were maintained and propagated at the SPF animal feeding standards under a 12-h dark/light cycle in a specific pathogen-free animal facility. Postnatal 20 male C57BL/6J WT mice and SCARKO mice were used for experiments in this study.

### Histology of Testis and Epididymis

Mouse testes and epididymis were dissected from P20 WT and SCARKO male mice and fixed in 4% paraformaldehyde (PFA) overnight. Then tissues were embedded in paraffin, sectioned into slides and stained with Hematoxylin and Eosin (HE). HE was performed as described before. After sealing with neutral gum, images were captured with microscope (Olympus, BX53, Tokyo, Japan).

### Immunofluorescent Staining

Paraffin-embedded testis sections were deparaffined and dehydrated as above. Then antigens were retrieved in 10 mM sodium citrate buffer (pH 6.0) in a microwave. Non-specific antibody binding sites were blocked with 5% BSA for at least 30 min at room temperature. Primary antibodies were diluted in PBS and incubated at 4°C overnight. The primary antibodies used were rabbit mAb AR antibody (ABclonal, A19611, 1:50). Slides were then washed and incubated with secondary antibodies [donkey anti-rabbit IgG-Alexa Fluor 488 (Invitrogen, A21207, 1:1,000)] for 2 h at room temperature. After three washes, testis sections were counterstained and mounted with DAPI-containing Vectashield (Vector Laboratories, H-1200) and observed under confocal microscopy (Zeiss, LSM710, Germany).

### Testis Sample Preparation

Single testicular cells were isolated using a two-step enzymatic digestion protocol described previously (Sohni et al., 2019). Testes from P20 WT or SCARKO male mice were dissected and decapsulated in PBS. Collagenase Type IV (Sigma-Aldrich, V900893, final concentration of 1.0 mg/mL) was added to the tubules and incubated at 37°C for 5 min with gentle agitation. The separated tubules were washed with PBS twice and followed by digestion with trypsin (Gibco, 15090046, final concentration of 0.6 mg/mL) and DNase I (Sigma-Aldrich, DN25, 10 ku/mL) at 37°C for 20 min with periodic vigorous agitation. The cells were filtered through a 40 μm cell strainer and pelleted by centrifugation at 600 g for 5 min at 4°C. After washing with PBS twice, dissociated cells were used for scRNA-seq.

### 10x Genomics Library Preparation

The sample is made into a single-cell suspension, with CountessII Automated Cell Counter for Cell counting and Cell viability measurement, the Cell activity is required to be higher than 80%, and the Cell concentration is adjusted to the ideal concentration of 1,000/μL and processed as described previously (Macosko et al., 2015). Briefly, the prepared single-cell suspension was combined with the gel beads containing barcode information and the mixture of enzymes, and then encapsulated in the “double cross” droplets of microfluid to form gel bead-in-Emulsions (GEMs). The effective GEMs include gum beads (prefabricated 10X primers), single cells, and Master Mix. Then, cell lysis and reverse transcription reaction were carried out in GEMs. In effective GEMs, 10X Barcode would connect the GEMs with the cDNA products, then the GEMs were broken and the oil droplets were smashed, and PCR amplification was carried out using the cDNA as the template. After the completion of the cDNA amplification, conduct quality inspection on amplified product (size of amplified fragment and output of amplified product). After the amplification products were qualified, the sequencing library was constructed. First, the cDNA was broken into 200–300 bp fragments by chemical method, eDNA was segmented, the end was repaired and A was added, and the cDNA fragment was screened. The P7 Adaptor connector was connected and the sample Index was introduced by PCR amplification. Finally, the cDNA library was obtained by fragment screening. After the library was completed, the database was checked. Illumina HiSeq sequencing platform was used for sequencing to obtain the sequencing data, and the subsequent data analysis was carried out.

### scRNA-Seq Data Processing

Basic statistics were made on the quality of the original reading fragments using FastQC. Then, Trimmomatic software was used to preprocess the Illumina pipeline reading fragment sequence in FASTQ format, which can be summarized as follows: First, remove sequences of low-quality reading fragments: use a 4-base wide sliding window to scan the reading fragments and cut them when the average mass of each base drops to < 10. Second, remove trailing low-quality reading fragments or N bases (mean mass < 3). Third, remove the adapter sequence: there are two ways to delete the adapter sequence. One is to align with the adapter sequence and remove the matching cardinality greater than 7 and the mismatch cardinality equal to 2. The other is to delete non-overlapping parts when the overlap base of reading fragments 1 and 2 is greater than 30. Fourth, reading fragments less than 26 bases in length were also deleted. Finally, the unpaired reading snippets are deleted. The rest of the reading that passed through all the filtering steps were counted as clean reading, and all subsequent analysis was based on that. As a result, we have successfully used FastQC to make basic statistics about clean data read quality.

### PCA and t-SNE Analysis

In order to simplify the gene expression matrix to its most important features, PCA of Cell Ranger was used to change the dimension of the data set from (Cell × gene) to (Cell × M), where M represents the principal component that can be selected by the user. The pipeline for reanalysis allows us to further reduce the data by re-sampling the cells at random and/or by dispersing the selection of genes across the data set. In order to realize the visualization of two-dimensional spatial data, the author uses Cell Ranger to transfer the data after DIMENSION reduction of PCA to the non-linear dimension reduction method T-SNE, and also reduces its running time by fixed the number of output dimensions at compilation time to 2 or 3.

### Identification of Specific Genes in Different Cell Clusters

In order to identify these genes whose expression is specific to certain clusters, we tested each gene and each cluster using Cell Ranger to verify whether the in-cluster mean is different from the out-of-cluster mean. To find genes that are differentially expressed between Cell clusters, we used Cell Ranger to perform a quick and easy method called sSeq (Robinson et al., 2010), which uses a negative binomial precision test. When the count was larger, it was then switched from Cell Ranger to the fast asymptotic beta test used in edgeR, which was run on each Cell cluster and compared to all other cells, and then generated a list of genes that were differentially expressed in each cluster relative to the rest of the cells. We used Cell Ranger to calculate the relative library size, dividing the total UMI count per Cell by the median UMI count per Cell. As with sSeq, normalization is implicit because the parameter of each cell library size is combined as a factor in the precise test probability calculation.

### Enrichment Analysis of KEGG Pathway and Gene Ontology Function of Differentially Expressed Genes

Enrichment analysis of KEGG (Kanehisa and Goto, 2000) and GO (Gaudet and Dessimoz, 2017) differentially expressed genes was performed using the cluster analysis Profiler R package, which corrected for gene length bias. KEGG Pathways and GO terms with corrected *P* < 0.05 (FDR < 0.05) were considered to be significantly enriched in differentially expressed genes. In addition, the author also used gene MANIA (Zuberi et al., 2013) in Cytoscape3.6 to reveal the interaction network between KEGG pathway and genes in GO functional annotation.

### Quantitative RT-PCR

Total RNA from WT and SCARKO mice was prepared with Trizol reagent according to the manufacturer’s instructions. Then RNA was reverse transcribed into cDNA using the PrimeScript RT Master kit (Takara, RR037A). RT-qPCR was performed with the SYBR^®^ Premix EX Taq^TM^ II PCR Kit (Takara, DRR041A) following the manufacturer’s instructions on the Roche Lightcycler 480 Real-Time PCR System. Data were calculated according to the Applied Biosystems comparative *Ct* method.

## Data Availability Statement

The datasets presented in this study can be found in online repositories. The names of the repository/repositories and accession number(s) can be found below: https://www.ncbi.nlm.nih.gov/, PRJNA756896.

## Ethics Statement

The animal study was reviewed and approved by the Ethics Committee of Peking University Shenzhen Hospital and animal center of Shenzhen PKU-HKUST Medical Center.

## Author Contributions

CC, QM, and SM performed the experiments and data analysis. GS and QL prepared diagrams and wrote the manuscript. CC and QM designed the project. YG and JY supervised the project and provided financial support. All authors contributed to the article and approved the submitted version.

## Conflict of Interest

The authors declare that the research was conducted in the absence of any commercial or financial relationships that could be construed as a potential conflict of interest.

## Publisher’s Note

All claims expressed in this article are solely those of the authors and do not necessarily represent those of their affiliated organizations, or those of the publisher, the editors and the reviewers. Any product that may be evaluated in this article, or claim that may be made by its manufacturer, is not guaranteed or endorsed by the publisher.
