# Supplementary figures and images for: Single-Cell RNA Sequencing Defines the Regulation of Spermatogenesis by Sertoli-Cell Androgen Signaling
Source: Front Cell Dev Biol. 2021 Nov 15;9:763267. doi: 10.3389/fcell.2021.763267 (PMC8634442; doi:10.3389/fcell.2021.763267)

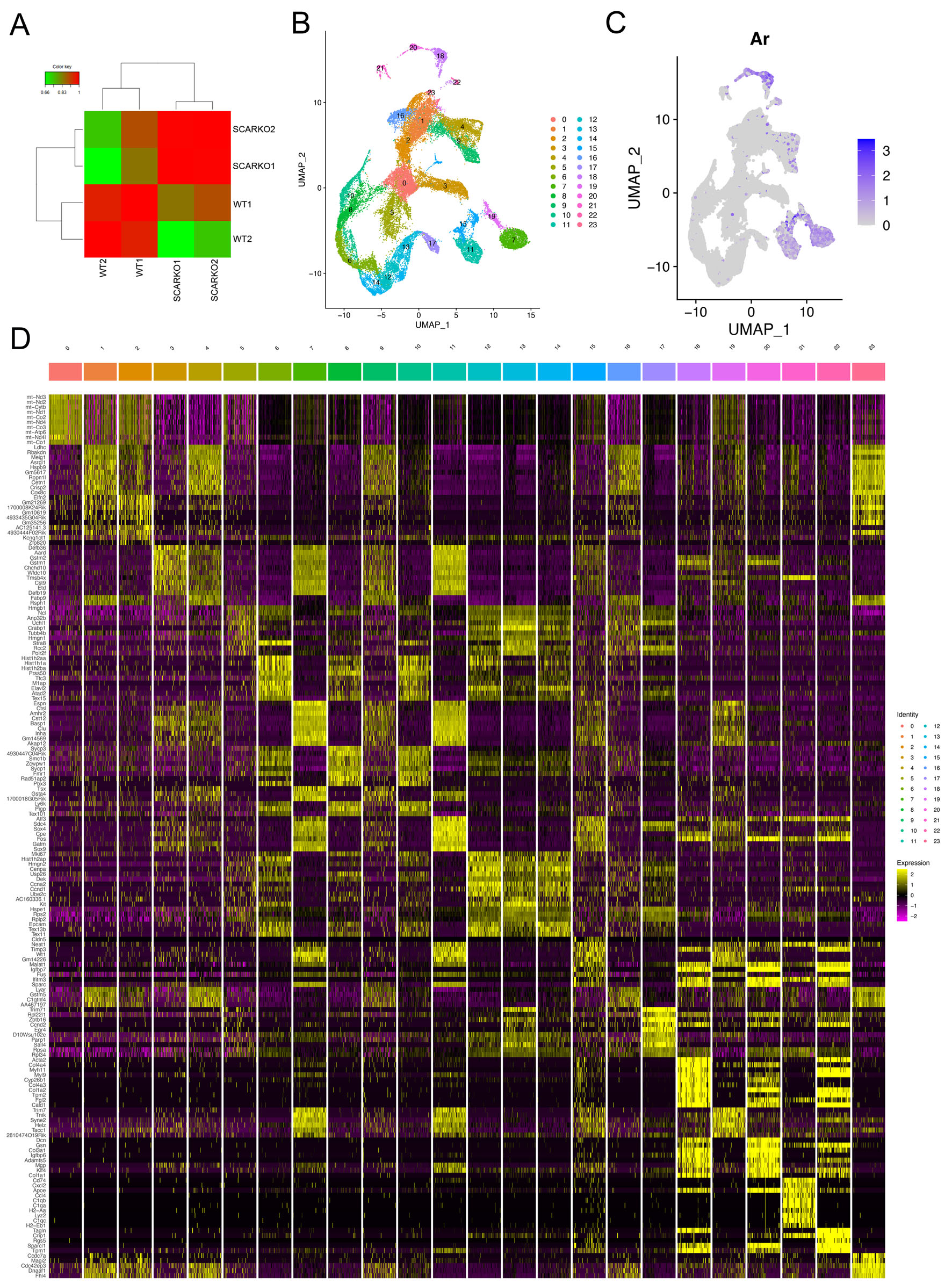

Supplement: Supplementary file 1 [file Image_1.JPEG]

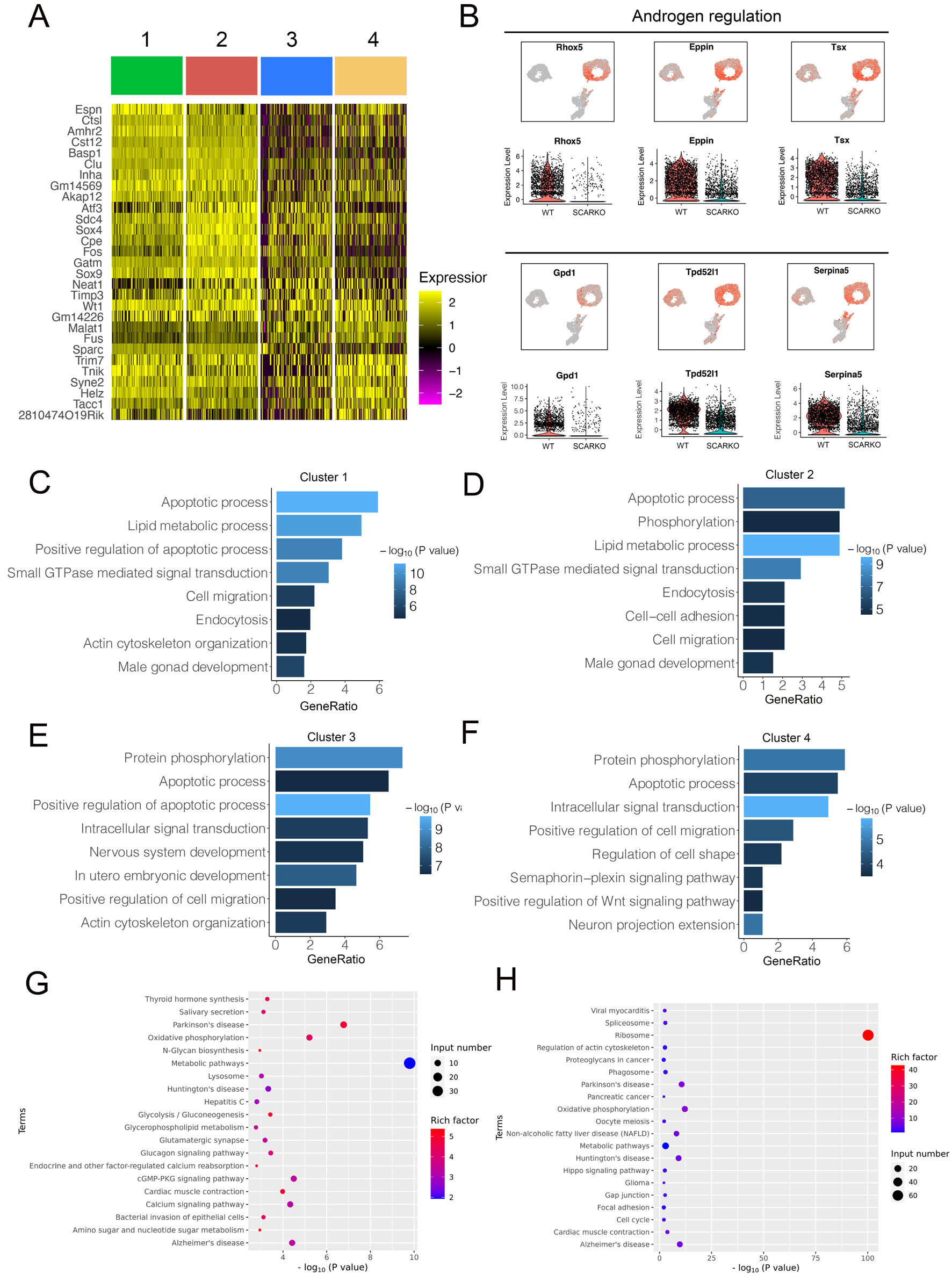

Supplement: Supplementary file 2 [file Image_2.JPEG]

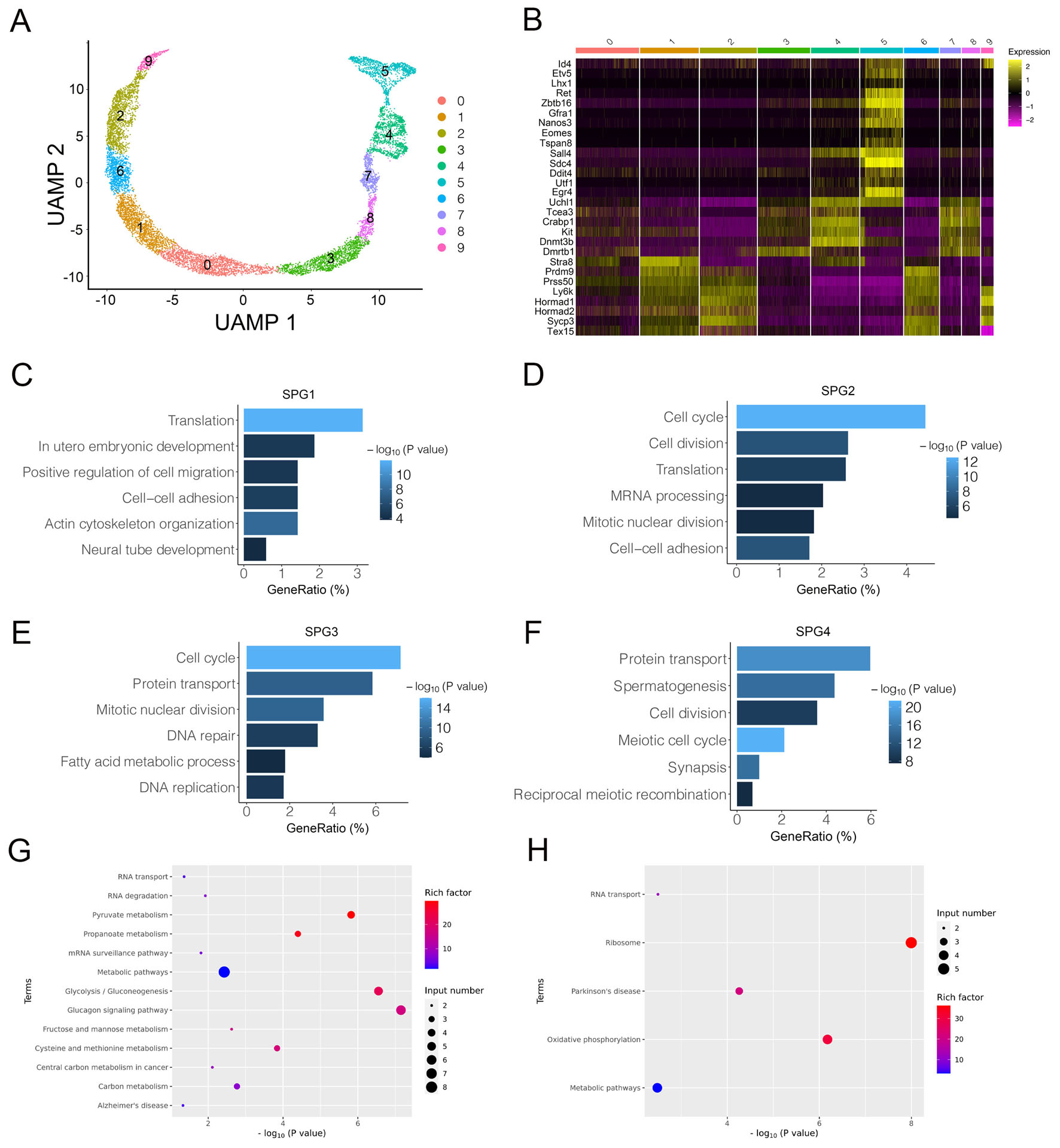

Supplement: Supplementary file 3 [file Image_3.JPEG]
